# Challenges in Implementing Antimicrobial Stewardship Programmes at Secondary Level Hospitals in India: An Exploratory Study

**DOI:** 10.3389/fpubh.2020.493904

**Published:** 2020-09-18

**Authors:** Philip Mathew, Jaya Ranjalkar, Sujith John Chandy

**Affiliations:** ^1^ReAct Asia Pacific, Department of Community Medicine, Pushpagiri Institute of Medical Sciences, Thiruvalla, India; ^2^ReAct Asia Pacific, Pharmacology and Clinical Pharmacology, Christian Medical College, Vellore, India

**Keywords:** antibiotic stewardship, AMS, healthcare, LMIC, infection, implementation, barriers, factors

## Abstract

**Introduction:** Implementing a sustainable and effective Antimicrobial Stewardship (AMS) programme in secondary level hospitals, in Low-Middle Income Country (LMIC) contexts, has numerous challenges. It is important to understand these challenges so that the stewardship initiatives can be tailored according to the unique requirements thrown up by these healthcare facilities. This study explores the experiences of implementing AMS in secondary level hospitals in the state of Kerala, India.

**Methods:** A qualitative study was planned to map the challenges in implementing AMS in the secondary level hospitals. Toward the end of the 1 year followup period, the nodal officers at each hospital were interviewed using a semi-structured interview guide. The in-depth interviews were transcribed and later subjected to content analysis using N-Vivo 11.0, a popular software tool used for qualitative analysis.

**Results:** Many physicians cite perceived patient satisfaction as one of the reasons for increased antibiotic use, as many patients consider antibiotics as standard of care. Also, the distance traveled by the patient and advancing age are factors which increase antibiotic use. The physician factors which determine use include empiric treatment needs, outbreak of diseases, absence of education programmes in antibiotic usage to fill in the knowledge gap and fear of litigation. The promotional activities by companies and antibiotics being a major source of income for small hospitals, affects use patterns. The factors which determine antibiotic selection includes conformism, experience of the physician, perceived resistance to certain antibiotics, emergence of specific diseases, and promotional activities related to antimicrobial agents. The challenges in implementing a sustainable stewardship programme is multifactorial. It includes competition between doctors, time constraints faced by physicians, absence of a champion, sub-optimal interdepartmental cooperation, absence of supporting facilities, dysfunctional regulatory systems, and unreliability of antibiograms.

**Discussion:** AMS in resource-limited setting is going to be a challenge, especially in terms of financing, access to technologies and capacity building. Political and regulatory willpower of international partnerships should be effectively harnessed for designing solutions for LMIC contexts. Also, models for stewardship from elsewhere should undergo an adaptation process before implementation in low resource settings.

## Introduction

Antibiotic Resistance has emerged as a global health problem, particularly with the advent of Healthcare Associated Infections (1). Antibiotic resistant organisms are capable of causing life threatening infections. The gravity of the problem has increased since research and development of newer antimicrobial agents has significantly diminished. Hospitals and health centers prescribe and dispense a lot of antibiotics, thereby increasing the selection pressure on bacteria to develop mechanisms of resistance; and this factor significantly contributes to healthcare facilities becoming hubs for development of multi-drug resistant organisms (2).

Antibiotic Stewardship (AMS) is often cited as a major strategy to rationalize the use of antibiotics and prevent emergence of resistance in various settings. AMS is a set of coherent activities that results in responsible use of antibiotics, with the definition of responsible use being context-specific and updated periodically (3). The role of AMS in a healthcare facility has been demonstrated through various studies (4–6). These studies show significant reduction in use of antimicrobials, expenditure associated with antimicrobial use and levels of resistance among various indicator pathogens. Any AMS programme should be seen as complementary to the Infection Prevention and Control initiatives as they have a shared objective and are synergistic (7).

AMS programmes in hospitals need significant funding support, trained human resource and political will. A robust level of implementation of stewardship measures in a hospital requires a committed team of experts; and the support of microbiology laboratories and hospital information systems (8). However, smaller hospitals face challenges in implementing AMS measures as they have difficulties in forming a multidisciplinary team, often have inadequate funding support and microbiology labs, if available, have minimal capacity with challenges in quality assurance (9). In the context of developing countries, most AMS programmes have been piloted in tertiary care healthcare facilities in urban areas; and very little effort has been taken to look at the feasibility of implementing these interventions in smaller hospitals or primary healthcare centers (10). The interventions that are feasible and effective in low-resource settings, may be different from those which has succeeded in larger hospitals situated in High Income Countries (11). Therefore, it is important to conduct contextual evaluation of the efforts to implement AMS programmes, with a focus on smaller healthcare facilities in Low and Middle Income Country (LMIC) settings.

## Methods

With the above issues in mind, a project was undertaken to develop an AMS model for rural secondary level hospitals in southern India. A total of 7 centers were recruited for the project and a start-up workshop was held for representatives from the hospitals. A doctor and a pharmacist from each hospital attended the workshop. The start-up workshop sensitized the participants about the various processes in AMS and hospital infection control. An exhaustive list of possible stewardship interventions which could be introduced in the hospitals were discussed during the workshop. There was general consensus that implementing a host of interventions at the beginning of the project would be difficult as many of them were not doable in those contexts and there were financial limitations. It was decided to implement one intervention each for the in-patient and out-patient departments of the hospital, and the interventions were selected through a group discussion aimed at creating consensus. For the outpatient sections, having an antibiotic algorithm for upper respiratory tract infections and intensive education on the same was selected as a doable intervention. Measurement of Days of Therapy (DOT) before and after implementation was selected as the evaluation tool. The DOT was assessed using prescription audit of 100 consecutive patients who are diagnosed to have upper respiratory tract infection. For the inpatient sections, considering de-escalation of antibiotics at 48 h after admission was selected as a doable intervention. Proportion of patients who underwent de-escalation of antibiotics, was selected as the outcome measure. The hospitals were provided continuous guidance and there was a provision to consult AMS experts from a referral center in case they faced any challenge during the period of implementation. A follow-up workshop was held 8 months after the initial event, to reinforce the learning points and to assess the success.

A qualitative study using in-depth interviews was undertaken to document the challenges faced by the key personnel, during the stewardship implementation process. The in-depth interviews were conducted using a semi-structured interview guide (Supplementary Material) prepared for the same. The interview guide was designed with the help of experts and inputs made through a thorough literature review. The study was approved by the Institutional Review Board of Christian Medical College, Vellore, India, and written informed consent was taken from all the participants. One person from each hospital, who was primarily responsible for implementation of stewardship, was interviewed for the study. Two hospitals refused to give consent and therefore 5 interviews were made for the study. The interviews were conducted in the local language and recorded. The recordings were transcribed and translated to English. The transcripts were entered into NVivo version 11, a qualitative data analysis computer software to do various levels of coding. The approach taken was to move from descriptive data to identify more abstract themes. It started with familiarization and subsequent indexing and categorization of data. After that abstraction was done identifying more analytic concepts, interrogating them for patterns of meaning.

## Results

### Profile of the Hospitals

Five of the seven hospitals were secondary level ones; while two of them were functioning in secondary and tertiary capacities. Since all of them are from the private sector, they are not formally classified into secondary or tertiary levels. For the purpose of the study, the classification was done on the basis of the kind of services provided by them. The profile of the hospitals are given in Table 1.

**Table 1:** Basic Profile of the hospitals.

**Hospital**	**Location of the hospital**	**Type of the hospital**	**Number of Hospital beds available**	**Average Number of outpatient visits per month (2018)**	**Average number of outpatient visits in Internal Medicine per month (2018)**	**Average Number of inpatients in Internal Medicine wards per month (2018)**
Hospital 1	Rural	Secondary	100	9000	1500	100
Hospital 2	Rural	Secondary	70	2500	1500	20
Hospital 3	Rural	Secondary/Tertiary	650	33,000	6,000	600
Hospital 4	Rural	Secondary	50	1700	1700	250
Hospital 5	Urban	Secondary/Tertiary	450	26000	5000	400
Hospital 6	Rural	Secondary	100	11000	3000	750
Hospital 7	Rural	Secondary	100	8000	2500	500

### Results of the Interventions

Quantitative assessment of the impact of the two AMS interventions were assessed at the end of the follow-up period. The data was collected by the nodal officers, from patient records maintained at the hospital. Both the interventions failed to show sufficient impact and overall details are given in Table 2.

**Table 2:** AMS interventions implemented in the hospitals.

**Patient care area**	**Description of AMS intervention**	**Measure of success**	**Source of Data for measurement**	**Impact**
Outpatient	Educational intervention for physicians to rationalize use of antibiotics with a standard treatment algorithm for patients with uncomplicated Upper Respiratory Tract Infections	Days of Therapy (DOT), measured before and after the intervention	Outpatient records of patients and copies of prescriptions stored in pharmacies	Failure, as the overall reduction in DOT was not statistically significant
Inpatient	Educational intervention for physicians to consider patients for de-escalation of antibiotic therapy, 48 hours after admission to internal medicine ward	Proportion of eligible patients undergoing de-escalation of antibiotic therapy.	In patient records and doctors order sheets for patients	Failure, as only less than 5% of the eligible participants had de-escalation of antibiotic therapy.

### Profile of the Participants

The participants were aged between 24 and 50 years of age; and were from diverse health professional backgrounds. All the participants had attended the two workshops and were directly involved in the implementation process of the AMS interventions in their hospital. The basic profile of participants is given in Table 3.

**Table 3:** Basic profile of participants.

Name^*^	**Age group**	**Current designation**	**Work setting**
Renji	30–39	Physician	Secondary
Suraj	30–39	Pharmacologist	Tertiary
Abi	20–29	Junior resident	Secondary
Mini	20–29	Clinical pharmacist	Secondary
Joby	50–59	Intensivist	Tertiary

* *Names have been changed for maintaining anonymity*.

### Reasons for Antibiotic Misuse

**Figure 1:**
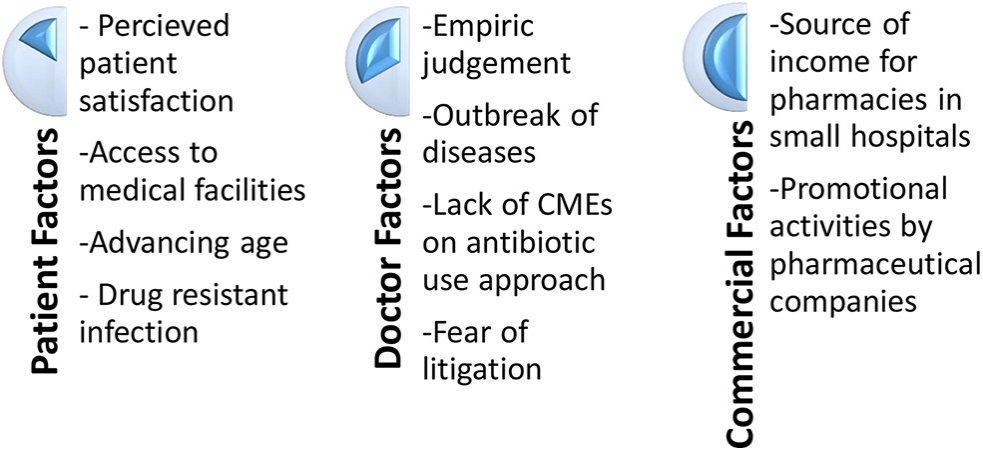
Factors for inappropriate use of antibiotics.

The first part of the study evaluated the various reasons for antibiotic misuse in their hospitals. The responses were clustered into different themes/nodes during the analysis phase, for better understanding and representational purposes (Figure 1).

#### Patient Factors

a) Perceived Patient satisfaction: Multiple participants said that many patients tend to view antibiotics as the standard of care in any kind of infection. They feel that patients will be satisfied only when they are prescribed antibiotics; though many patients may not verbalize it. Antibiotics are often perceived as the “*go to weapon in the arsenal of a physician” [when the patient is] seeking cure as fast as possible from [communicable diseases].”*

“*[There is this] perception that if you immediately use an antibiotic, you can save a patient or the patient can do better.”*

The fear of losing the “*allegiance or loyalty of patients”* is cited as another reason for prescribing antibiotics. “*Basically it is like one needs to […] make patients happy and […] send them off satisfied. […] longer the course of the disease, less happy and satisfied [patients are]. [Antibiotics] have definitely shortened the course of the disease”*.

But the clinical pharmacist who was in the study seems to have a different opinion when compared to other participants who were medical doctors. She feels the notion that patients will not be satisfied without antibiotics, is just a “perception” that doctors tend to harbor. She feels that the reality might be different. For this she quotes a small survey she had conducted among OP patients in which apparently 70% left the decision to the discretion of the doctor and did not demand for antibiotics. According to her “*doctors prescribe antibiotics assuming that, this is what patient expects, but the reality may be contrary. For among those who had come with a throat pain probably a 5% may require antibiotics. But doctors end up prescribing antibiotics to all. There is a lot perception issues involved. I feel if we educate patients properly they will definitely come around. Nobody likes to take medicines.”*

b) Accessibility to medical facilities: Many a times, it appears that the distance between patient home and the medical facility plays a very important role in deciding whether antibiotics should be prescribed or not. Especially when it involves “*elderly patients who will have difficulty in getting a bus and coming back [with] no people to accompany them.”* Sometimes “*the person may be the [sole] bread winner of the family. During consultation, while listening to the travails of a rural family, [sometimes] certain factors would become apparent which tell you that admission might not be possible for the patient.”* When faced with such situations, I felt that doctors would start antibiotics pre-emptively. It appears to me that they are often guided by notions as in the patient's (the bread winner) condition may get worse pushing the whole family into starvation.

c) Advancing age: In the state of Kerala, India, the population is aging fast. Though it is a sign of social development, one physician identifies this as one of the risk factor for increase in antibiotic use. His hospital mainly caters to the old age population as it is situated in the most aging part of Kerala.

“*This is an aging population. Though this is a developed part of Kerala, it is really an aging population. 80% of our inpatients [because of the nature of their illness] require antibiotics and it is not really dominated by commercial considerations.”*

d) Likelihood of drug resistant infections: One of the physicians also cited increasing drug resistance as a reason for antibiotic misuse. Sometimes antibiotics are prescribed till there is a clinical response, without taking into account the natural history of disease.

“*We admit the patient, we start them on the antibiotic, we see a response and we stop the antibiotic and discharge the patient. If do not see response we continue the antibiotic until we get a response.”*

#### Doctor Factors

a) Empirically after clinical judgement: Many doctors justify antibiotic use by quoting clinical signs and symptoms in the patient. Two participants explained how they would choose antibiotics empirically, by citing signs and symptoms of commonly seen conditions in their day to day practice.

“*You educate the patient to come back, if they have purulent sputum. They come back telling that they have purulent sputum in the morning. [The sample may appear to be clear at the time of examination]. But this clue is enough for doctors to start on antibiotics”*

Laboratory investigations are often done to make decisions evidence based. However, there is no uniformity regarding which is the investigation that needs to be considered as the standard to initiate treatment. Investigations itself has been brought out as a cause for increase in antibiotic use. It is almost like treating lab results than symptoms.

“*Some people are obsessed with things like CRP and if the CRP is high you have to give an antibiotic or if you have sent for [blood] counts and [if counts are] high you give antibiotics. Otherwise patient may be asymptomatic.”*

b) Outbreak of diseases: Outbreaks of diseases, especially in the monsoon season, is cited as a factor which increases antibiotic use.

“*Outbreak of diseases definitely increase the antibiotic use. Like we do have viral epidemics coming on and off”*

The Clinical Pharmacist expressed the view that “*[Doctors] shows this tendency to start antibiotic as a prophylaxis. [Once] I saw few drugs with a patient, one of them was Amoxicillin. She got it from government hospital. Apparently she was told to take it whenever she felt febrile. There are so many people who carry around medicines like this.”*

A physician expressed the view that presence of “*underlying comorbidities make patients really sick”* thereby increasing the possibility of using antibiotics. The pharmacologist was of the opinion that it is not the outbreak of diseases, but the emergence of new diseases that prompt use of antibiotics.

“*Because of emerging new diseases […] the perception is that with an immediate use of antibiotic, one can save a patient or the patient can do better.”*

c) Lack of Continuing Medical Education (CME) sessions on antibiotic usage to fill the knowledge gap among prescribers: According to the junior resident, senior doctors continue to follow what they were taught while they were students for the rest of their life.

“*Antibiotics were on the rise and the challenges of resistance and all probably might not have developed during the early 80s and 90s. So those who have completed their primary medical education during early 80s and 90s themselves believe that antibiotics are the highest standard of care.”*

He also noticed that doctors who lack academic direction prescribe antibiotics out of habit, and without any scientific foundation. “*A lot of physicians who are practicing in and around this area are not really academically oriented. So they are not aware and they habitually prescribe antibiotics. So there is a knowledge gap.”* He feels that if we are giving antibiotics on the fifth or sixth day of illness when the conservative management does not show any improvement, patient may feel that the doctor had withheld *the standard of care* thereby losing their confidence. To us that statement showed his own poor understanding/misunderstanding about antibiotics.

The Pharmacologist felt that even if junior doctors know things better, it will not be possible for them to defy seniors' orders as the medical system tends to be very hierarchical. “*Because sometimes what happens is even though the junior doctors withhold the antibiotics, they get scolding from the senior saying why did you withhold the antibiotics.”*

The clinical pharmacist quoted lack of proper awareness regarding spectrum of each antibiotic as the reason for double coverage and antibiotic over use. According to her, often the source of information about antibiotics is the briefing by medical representatives.

“*I feel they are not very aware about the spectrum of each antibiotic. […] In a way it is knowledge gap, they do not have a proper understanding regarding these things. Their understanding is mainly based on what medical representatives tell them [who often] teach things like if you are giving Penicillin, you have to prescribe Clarithromycin along with it. […]. Many fixed dose antibiotics are getting prescribed because of medical representatives.”*

d) Fear of litigation: The clinical pharmacist noticed that more than the poor social background it is the fear of litigation which doctors suggest as the reason to prescribe antibiotics.

“*Their argument was if the patient comes back with surgical site infection people will only blame the surgeon”*

During her interaction with doctors she also noticed that doctors fear that “*[if] they sent of patients without antibiotics, they would come back with infection, mainly because of the poor hygiene and the poor economic status of Indian patients”*.

#### Commercial Considerations

a) Source of income for pharmacies in small hospitals: The junior resident, who is working in a secondary setting that mainly treats acute conditions, felt that cutting down on antibiotics will tap into their pharmacy income. According to him, “*at least 30 to 40% of [their] pharmacy income comes from antibiotic [sale].”* The category and level of hospital may be a factor when it comes to commercial consideration at the institutional level. For small hospitals which mainly handle acute conditions, antibiotics sale is a major part of their revenue. As in the case of the junior resident's hospital, “*[…] we do not really treat a lot of other illnesses. Its usually upper respiratory tract infections and urinary tract infections that come here. Majority of the income is from antibiotics. So there is a definite commercial consideration.”*

b) Promotional activities by pharmaceutical companies: Promotional activities by pharmaceutical companies also play a role in increase in antibiotic use. As mentioned earlier, sometimes their overzealous promotion results in double coverage. The clinical pharmacist felt that medical representatives, while introducing a new antibiotic to a doctor, might not have explained its entire spectrum. This will invariably lead to doctors writing another antibiotic unknowingly leading to double coverage. “*For example, suppose [doctors] are writing an antibiotic suggested by a representative and suppose it has got anaerobic coverage [which doctors] might not have checked. They will start metronidazole also, saying anaerobic coverage.”* Though we felt the blame cannot be put entirely on pharmaceutical companies, their promotional activities could be lopsided by projecting only the spectrum which they are interested in.

### Factors Guiding the Antibiotic Selection

**Figure 2:**
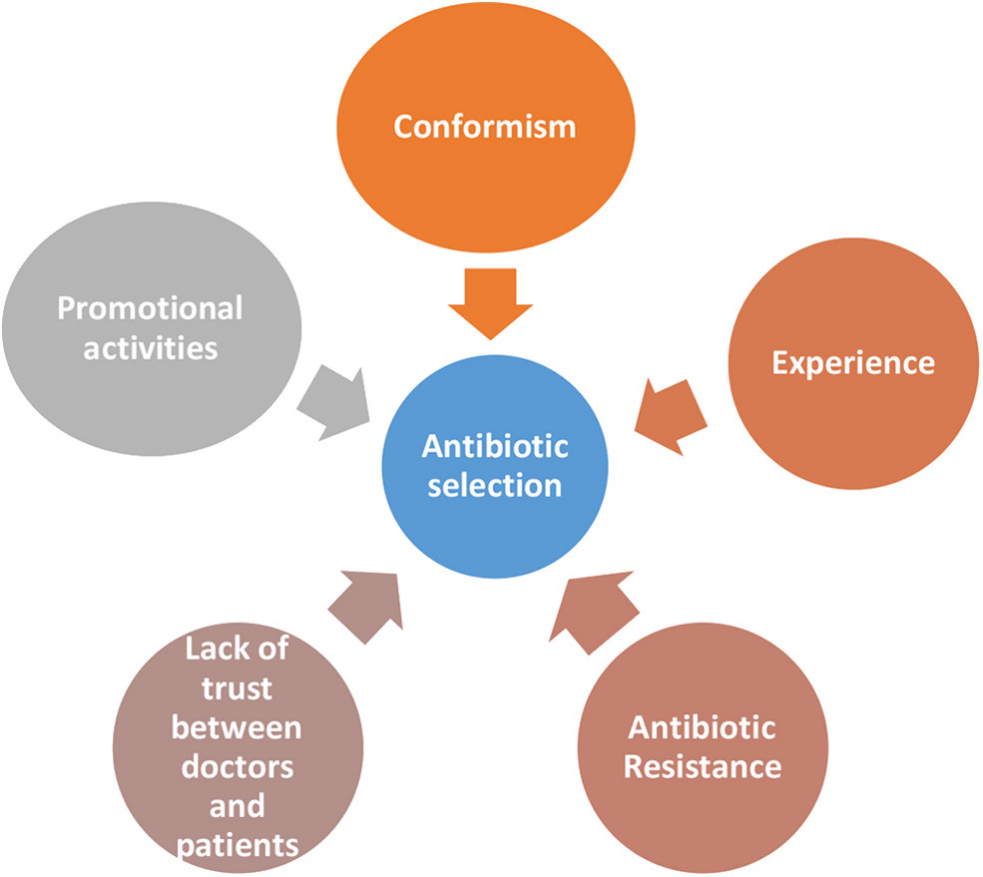
Factors guiding antibiotic selection.

The second part of the study dealt with factors which determine selection of particular antibiotics in secondary care settings (Figure 2). All participants named amoxicillin-clavulanic acid combination as the most commonly used antibiotic in the out-patient setting. Other antibiotics named were cefixime, azithromycin, and levofloxacin. The clinical pharmacist also highlighted that “*Plain amoxicillin is hardly available now a days.”* In the inpatient setting the commonly used first line antibiotics were third or fourth generation cephalosporins like ceftriaxone and cefaperazone which are commercially available in combination with β lactamases like sulbactum or tazobactum. One of the physician quoted an interesting tagline that was common among his colleagues, “*fever is not a sign of ceftriaxone deficiency”* to explain the extent of use of higher generation cephalosporins. This is often escalated to piperacillin and meropenem. The clinical pharmacist also mentioned that “*[the oral] linezolid is very commonly used by surgeons”*. She also feels that “*because of side effects, […] the use of quinolones have decreased considerably*.

#### Conformism

It appears as though most of the time doctors go by what everyone else is practicing than by evidence. We could not but help notice that all participants irrespective of whether they were working in a secondary or a tertiary setting quoted almost same antibiotics, when asked about commonly used antibiotics in in-patient and out-patient settings.

“*The antibiotic that was used by default [by everybody] was ceftriaxone. Now I have read somewhere fever is not a sign of ceftriaxone deficiency. […] invariably everyone uses ceftriaxone I think.”*

According to one physician, junior doctors who had just graduated, would blindly follow what seniors were practicing and would soon habitually acquire the same prescribing habits. The clinical pharmacist has also noticed a similar practice.

“*Suppose a doctor introduces a new brand, I have noticed that everyone will be writing that brand for anything and everything.”*

#### Experience

One physician shared the conversation he had with a colleague about the reason for prescribing fourth generation antibiotic for inpatients. The explanation of the colleague was “*there are lots of resistant gram negative bacilli in the community; we do get such cases. So in order to cover that I am starting this [cefaperazone-sulbactum].”* He however did not mention about how he had learnt about the presence of resistant gram negative bacilli in the community. As the junior resident puts it, “*the hospital system is structured in such a way that [usually] primary admitting physicians have their own protocols and policies, which are not really evidence based or validated”* and everyone just blindly follows it. One of the participants was from a hospital, where systems are in place. In that hospital culture sensitivity testing was done in many cases before starting the antibiotic.

#### Antibiotic Resistance

One of our participating physician feels that it is difficult to get antibiotic naïve patients, especially in a tertiary center. So they are forced to use higher antibiotics. He has also noticed that the resistance levels are higher than before and feels that excessive antibiotic use in non-medical sectors could be playing a role.

“*Previously when a patient gets referred from primary or secondary care centers we could manage them with carbapenams. But now when a patient is referred from a secondary center after 3–4 day hospital stay, the initial respiratory and urine samples are resistant to carbapenams. […] its excess use in farm sector, it could be playing a role.”*

#### Lack of Trust Between Doctors and Patients

According to the junior resident, this factor also plays a role in the selection of antibiotics. “*As patients become more and more aware of the possible legal support, [doctors fear that] they may use it […] for their own monetary benefit; the chances of physicians going for the big guns are higher.”* We felt that there is a growing lack of trust between doctors and patients, prompting doctors to go for defensive practices by prescribing “higher” and more “critical”; antibiotics.

#### Promotional Activities

The choice of antibiotics is often influenced by promotional activities by medical representatives. The clinical pharmacist feels that the doctors practicing in rural areas are very receptive to these promotional activities, as it may be their only source of information.

“*Their understanding is mainly based on what medical representatives tell them. They teach things like if you are giving penicillin, you have to prescribe clarithromycin along with it.”*

But the intensivist feels it is alright to promote certain companies since money is essential for conducting CMEs and other programmes in the hospital and this is the best way to raise money for the same. According to him, “*the unholy nexus exists, [doctors] need to know where to draw a line, and it is [often] difficult.”*

### Stewardship Measures Implemented/Planned

All participating hospitals attempted to implement two stewardship measures, which was decided during the start-up workshop.

One secondary level hospital managed to document and quantify prescribed antibiotics. These measures were taken to get “*an idea about the actual antibiotic use […] previously [in some hospitals], there was no way of identifying what antibiotics were being used. So at the end of the period we started getting an idea of the actual antibiotic use. [… with this] we managed to bring down the antibiotic use, as in every upper respiratory tract infection (URI) getting an antibiotic to almost no URI getting an antibiotic in the first 4 or 5 days. Even without having a proper formalized protocol, we were able to bring in changes in the system by means of an informal mechanism.”*

Preparing an antibiogram was an initiative by another hospital which is in the process of creating an antibiotic policy. The primary motivation behind this however appears to be an imminent National Accreditation Board for Hospitals & Healthcare Providers' (NABH) inspection and this makes its implementation a challenge. NABH is a board for quality control to maintain healthcare standards for hospitals and healthcare providers.

“*Because of NABH, hospital does not have any other choice, but to create an antibiotic policy. But I am not sure of the implementation part of it. I have a feeling that the policy will just remain in paper and will not get implemented.”*

The well-established center, which was already NABH accredited, already had a stewardship system in place. After attending the workshop they were better equipped to implement them.

“*They have developed area wise antibiograms as in in-patient, out-patient and Intensive Care Unit. Based on that we have updated antibiogram, using last 6 months data. Accordingly, we are revising our antibiotic policy and our prescriptions will be according to that. Restricted group of antibiotics are there that needs pre-authorisation, but that is not possible always. What we practice here is that we review it within 24 hours and stop if found unnecessary; a feedback will be provided when stopped. [So] there is no pre-authorization, but there is a check. Secondly, we are doing de-escalations also, […] may not be 100% time, but definitely in 50% of cases. The cultures are being sent routinely before starting antibiotics. […] so even if antibiotics are started the infection control team have proof […] for changing starting and stopping antibiotics.”*

Almost all participants mentioned doctors as main hindrance in establishing AMS in their facilities. “*If I get a better support from the management, from senior people, even from my department […] that will be a great achievement.”*

For hospitals which were in the process of applying for or had got accreditation, the management was very supportive of this initiative; otherwise the hospital management also tried to resist such change along with doctors.

The medical sector is very hierarchical in nature. Therefore, once the doctors were convinced, there was hardly any resistance from other staff as in pharmacy, nursing or microbiology toward this initiative.

### Challenges in Implementing Stewardship Program

**Figure 3:**
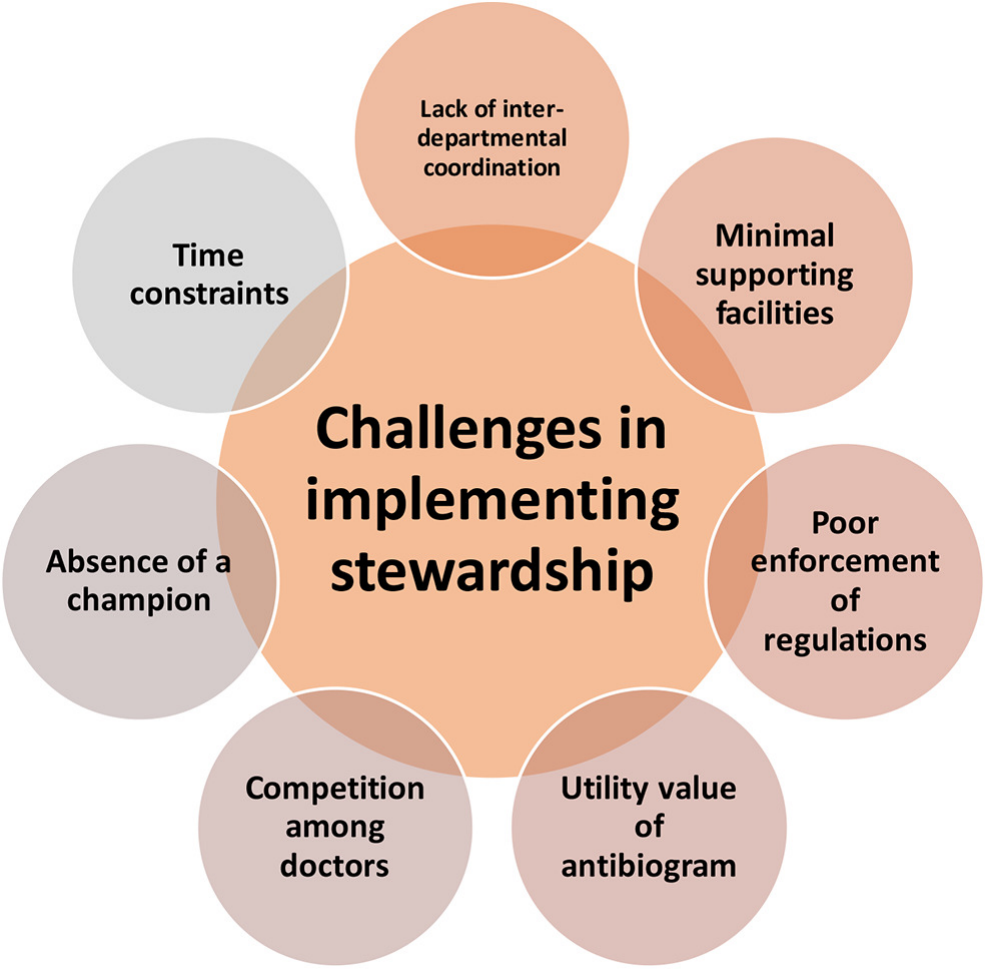
Challenges in implementing a stewardship program.

All the participants cited multiple challenges in effective implementation of a stewardship program in the hospitals. It was interesting to note that the challenges were in the form of systemic issues; and not financial constraints or lack of mandate (Figure 3).

#### Competition Among Doctors

It is difficult to start AMS initiatives in isolation as there is a fear of losing patients. As one *physician* puts it: “*When a policy is made it has to be universal so that the protocol is standardized irrespective of the doctor. In such case the hospital will not lose patients. But if the patient feels that he will get a “better treatment” [in this case easy availability of antibiotics] from another doctor […], the whole thing will get skewed […] the physician gets a bad reputation for withholding “good treatment.”*

#### Absence of a Champion

It appears as though the participants who have ventured into stewardship programme are not motivated enough to pursue it till the end. They tend to give up easily especially when they fail to convince difficult colleagues. Often, the reasons cited are very trivial.

“*The brighter one is with his/her studies, it is difficult to change convictions. Because he/she will have reasons for everything.”*

“*Since our hospital is situated in a remote place it is quite difficult to get doctors to work in our institution. So we are really scared to impose something on them as we are scared of losing them. […]. We have a floating doctor population. […] one cannot expect them to obey you.”*

As the pharmacologist has put it, many a time doctors could not see beyond the narrative “*they will lose their patients and if something happens then they will be held responsible for it;”* and hence should try every last weapon in the “*arsenal.”* Those who have taken up this mission should be convinced themselves to make others write responsible antibiotic prescription. In two of the participating hospitals, responsibilities for stewardship were given solely to a clinical pharmacist or to a junior most doctor. This appeared to be quite an overwhelming task. In fact the clinical pharmacist specifically mentioned “*the need to identify a champion who could lead.”* She also feels that a champion should be someone senior with good clinical exposure and academic standing. It has to be senior because as the junior resident puts it, “*the medical education system in India or Kerala does not really train a doctor to work independently when he/she graduates out from a medical college. Most juniors, after under-graduation, attach themselves to a hospital or a doctor and learn from their experiences. Often it is their habits that get pushed on to a new doctor and the system does not allow anyone to question one's senior.”* Even a person from an allied health field, as in a clinical pharmacist, cannot run the show. According to the clinical pharmacist, by asking questions “*our relationship [with doctors] will get affected. It is a hierarchical system because of which other staff in the medical field cannot question a doctor's decision. I think there is a need for culture difference in medical practice. In western countries I feel there systems are in such a way that, people like us can question a doctor- a professional to another professional- but that's not the system that's is prevailing here. […] there is a lot of ego issue involved. Doctors would be like… how a nurse could question me! They will not take it in a professional way.”*

Perceived clinical exposure is also very important because suggestions by clinical pharmacists often get ignored as “*textbook knowledge”* and are overthrown by “*experience.”*

“*They do not want to change from the style they developed. It is not exactly evidence based. I would say its eminence based. […] our medical superintendent is an ophthalmologist. His experience with handling antibiotics is much lesser than other seniors in the hospital as he deals with only few specific conditions. So others will not accept his view points or will over rule his decisions.”*

Doctors often raise concerns like “*if we send of patients without antibiotics, they will come back with infection, primarily because of abysmal hygiene levels and poor economic status of Indian patients. If the patient comes back with recurrent infection, doctors will be blamed. […] their justification is not always evidence based.”* I feel that only a person with sound academic basis will be able to address these issues scientifically and thereby convince doctors.

#### Utility Value of an Antibiogram

The clinical pharmacist feels that it is difficult to get an antibiogram which is a true representation of the resistance pattern in the community. She attributes it to the empirical treatment with antibiotics without sending for culture sensitivity. “*The culture is usually sent only when the patient becomes very serious, when things become very difficult to manage. […] Whenever there is an IP admission, the patient will be started on an antibiotic on the 1*^*st*^ *day of admission, usually on ceftriaxone. If you are sending a culture on day 1 of admission its report will come only after 3 days. By then three days of antibiotics have already been given. So they do not usually send for culture. Only if the disease does not respond to the antibiotic already written, they will send for a culture. Because of these things I feel our antibiogram is not very reliable. […] A patient who is already on an antibiotic, will usually show sensitivity only toward high end antibiotics. So if the doctors choose to treat looking at our antibiogram, they will have to choose all high end antibiotics.”* She also brings out another interesting point. Even if the antibiogram shows sensitivity to 1st or 2nd generation cephalosporins it is not of any practical use. Cefazolin, a recommended pre surgery prophylaxis is not available in the market. We found this to be an important point to be noted. After putting so much time, energy, money, and material for stewardship exercise, if the drugs required are not freely available are not freely in the market, the whole exercise is futile. This appeared quite ironical because antibiotics that actually need to be preserved can be procured easily through alternative methods with doubtful legality. “*There is a place in [city name] called “marunnu (medicine) street.” You go with the money and tell them I need pseudopenam—Meropenam in any name. You give whatever money they are asking. They ask you to comeback after few days and when you return you will get the licensed brand in your hand!!!”*

#### Poor Enforcement of Regulations

There is no point having a policy or programme targeting only the hospitals since as *the Pharmacologist* puts it “*when you do not give [patients] an antibiotic they will straight away approach a pharmacy where the pharmacist will supply them whatever they ask for or medicines which the pharmacist feels appropriate.”* Or else as the clinical pharmacist says “*[people] will keep old prescriptions with them and buy same medicines using those prescriptions when they develop similar complaints.”* Either ways people have other modes of getting medications. How to tackle this issue without making antibiotics inaccessible to those living in remote places is very challenging.

#### Time Constraints

One physician felt that implementation of stewardship measures like pre-authorization would be difficult especially in a crowded out-patient departments. “*…in out-patient department we have lots of patients coming in at a time. So it might take lots of time and patience from the side of the doctors to implement it.”* We felt that in a private setting, it is all about the profit one brings in, which is related to the number of patients seen by the doctor. Usually there will not be any cut offs for the number of patients one should see. “*Number of patients depend on patient satisfaction. […] patient is satisfied only when he/she thinks that the doctor has given him everything and hasn't withheld anything. If this is a tertiary hospital and [the doctor] did not prescribe what the patient wants, the likelihood of patient satisfaction would be higher. Patient perceives that a tertiary hospital knows what is best” says the junior resident*.

#### Lack of Inter-departmental Co-ordination

We felt that for implementing measures like stewardship, a sound management structure should exist. In the junior resident's experience no one is accountable for medicines that get intended to a pharmacy. “*Nobody is really accountable. Pharmacist does not know why he is ordering these antibiotics. The administrator does not know why pharmacist is asking for these set of medications. […]. The doctor would want to intend all medications which they want to prescribe.”* The system is too chaotic that it leaves many loop holes to by-pass it.

#### Minimal Supporting Facilities

Pharmacies in primary and secondary hospitals are not usually computerized. So it is often difficult to do quantification and documentation of antibiotics used as it has to be done manually. Even to make an antibiogram, participants have to manually go through all microbiology records to follow up sensitivity patterns over past 6 months or 1 year, which is a very tedious task. Some smaller facilities will not have a microbiology facility to handle cultures. This often prompt doctors to outsource culture sensitivity to independent laboratories outside, which will in turn prolong the reporting time.

“*Since it is outsourced, it is more expensive and takes more time for the results to come. […] Usually if it is sent at the beginning of the week and if there are no strikes in the state during that week (strikes occur quite often these days… (laughing), we will get the report within 4 days. Otherwise it will might take upto a week.”* says the junior resident.

### Sustainability Measures

**Figure 4:**
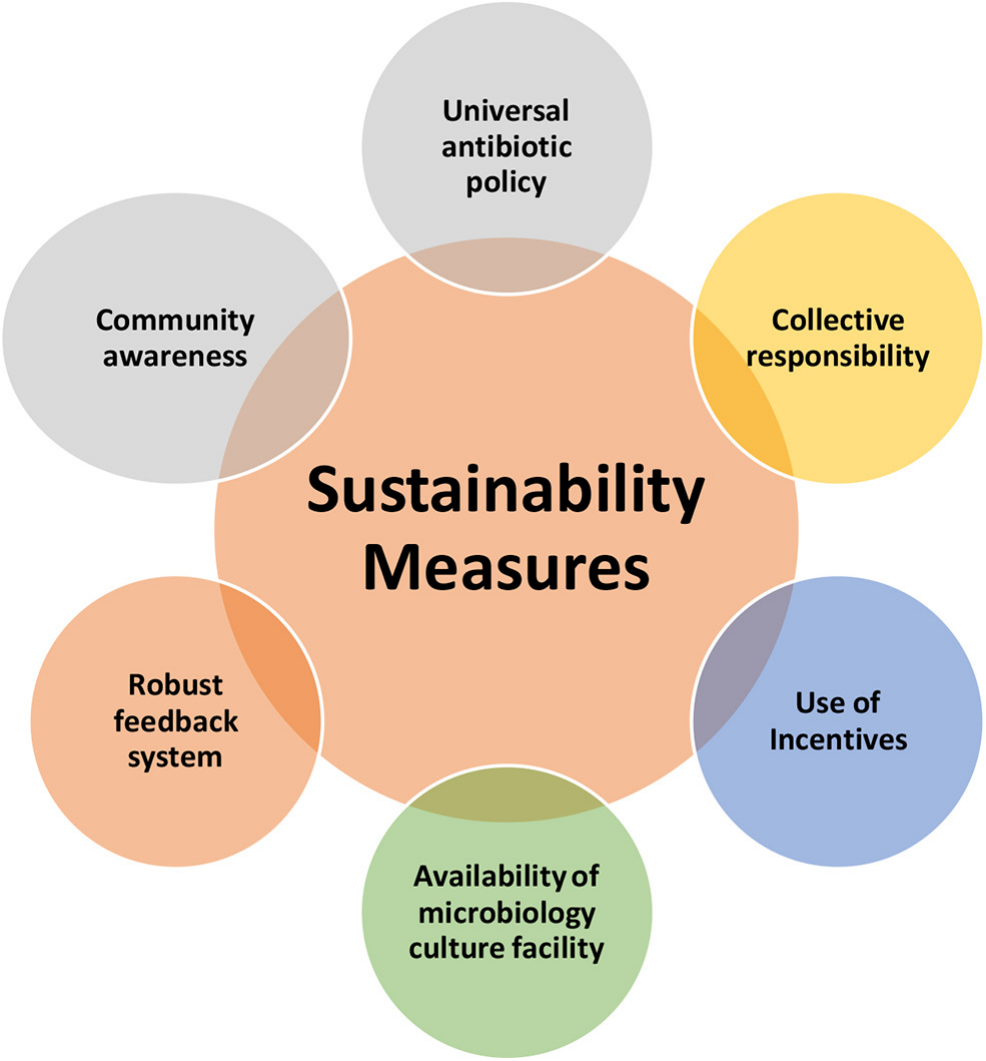
Sustainability measures for antimicrobial stewardship initiatives.

The semi-structured interviews also discussed about the possible sustainability measures, which can ensure the long term success of the AMS initiatives (Figure 4). The discussions were based on the sustainability measures within the context of secondary level hospitals in low-resource settings.

#### Universal Antibiotic Policy

We feel that it is important to work at the governmental level to ensure an antibiotic policy in all hospitals. This will alleviate the concerns of small hospitals as rightly put across by one physician “…*what is the effectiveness of implementing stewardship in isolated smaller settings when the nearest competitor is providing them without any restrictions.”* It may also give a head way to other facilities.

“*I feel that it will greatly augment our activities if the government steps to put in place policies which are practical and executable. Then the implementation will be much easier. We will not be having mountains to climb here.”*

The policy should also specify the stand on reserved drugs as in Linezolid or drugs which are kept as last resort as in carbapenems or polymyxins with respect to their market availability, sales and who should prescribe.

#### Use of Incentives

One suggestion was incentives in the form of award or title as in “This hospital uses antibiotics responsibly” will give a moral boost to hospitals. It will also help in improving the awareness among patients.

“*I do not think patients will feel like they need to get more antibiotics and run away to another hospital. I feel patients will think that these people are in right direction and will support us” says one physician*.

#### Availability of Microbiology Culture Facility

It appeared that to get a reliable picture of local antibiogram, all hospitals should have access to culture facilities. Secondary hospitals may not always be able to afford a microbiology facility. The junior resident came up with an interesting idea to tackle this. “*I think attaching a secondary hospital with a tertiary hospital may help. As in 10 secondary hospitals report to one tertiary hospital might actually help, where the tertiary hospital looks at the microbiological profiles of the area or patients.”*

#### Robust Feedback System

It is important to ensure feedback to doctors after conducting audit or any other stewardship measures, so that it will act as a self-assessment or a learning method. As one of the physicians puts it “*this measure is particularly easy to be implemented in a small setting, where the number of doctors are less.”*

#### Collective Responsibility

Transforming this issue from an individual conviction to a call for systemic change appeared to be a lynchpin. One suggested way to accomplish this is to identify a “champion” who “*can habituate the hospital […] to use antibiotics responsibly, so that the stewardship methods sustain. The key is about how to habituate the system”* says one of the physicians. As the clinical pharmacist rightly puts it, “*just giving doctors training will not help. It is not the awareness that is lacking, I think it is more of behavioral change is what that is required. Attending awareness programmes do not mean that they have accepted it. They will just come listen and go. Having a ‘champion’ may help. At least one person should be there. Otherwise if we can form a team, as in an antibiotic stewardship committee, which can include seniors, clinical pharmacists etc. something similar to hospital infection control committee, I think that also will be helpful. We need to identify a champion who could lead such a committee, I think then there will be a palpable change.”*

#### Community Awareness

For the responsible use of antibiotics, the consumer needs to be educated. According to the junior resident “*if one can highlight the bad effects of irresponsible antibiotics use in a way as powerful as the “smoking kills” advertisement, the patient attitude may change. The messaging should be structured carefully*, he continues, *in such a way that people should develop an individual ownership toward antibiotics and start using it responsibly.”*

## Discussion

AMS efforts have shown to reduce the utilization rates of antibiotics and the expenditures associated with these medicines. Studies have also shown significant reduction in the levels of resistance toward antibiotics included as targets of the programme, though this trend was not consistently recorded everywhere (5). However, even in centers with established AMS initiatives, the physician prescribing behavior is not optimal. Certain studies have shown that doctors tend to continue with the antibiotics, even when laboratory results rule out a bacterial infection (12). This shows the need to have a consistent programme which has a continuous training component.

Implementing stewardship programmes in smaller hospitals is a real challenge, especially in LMIC. Cost and human resources are the main barriers in implementing a successful AMS programme in these smaller hospitals. The traditional reasoning about “return on investment” does not really apply to the smaller healthcare facilities and community health centers. This is because most of such centers are standalone facilities and cater to only ambulatory patients. Also, it is difficult to obtain validated local data on antibiotic use and resistance patterns; and this makes the case even more complex (13). However, other models have demonstrated that AMS programmes can be implemented in rural and remote hospitals with minimum investment, as most of the day-to-day work can be carried out by non-experts. Capacity building of the existing human resources and support from the administrators are key components of a successful AMS model (14). But in the follow-up stage of our study, it was found that the two interventions implemented across the selected hospitals failed to have a significant impact. It is possible that the model was unsuccessful as prescriber behavior is more complex than anticipated and simple educational interventions may not be effective in bringing about a sustainable change in behavior (12). Also, it may be that the intervention and the follow-up period was short; and the timeline of implementation was insufficient to create a feeling of ownership among the prescribers.

Lack of a uniform national policy strictly enforceable in all healthcare facilities may affect the prescribing patterns of doctors. The patchy implementation efforts make it difficult for campaigners to convince doctors about rational prescribing of antibiotics and prescription autonomy (15). The rampant use of antibiotics without prescriptions is also affecting the prescription autonomy of the physicians (16). As one physician remarked during the interview, if they do not prescribe any antibiotic, the patient will go to the nearby pharmacy or the doctor next door and procure them. The increased competition between doctors and healthcare facilities, in some contexts in LMICs, can also increase the antibiotic prescription rates. This has been observed in studies done in high income countries, where prescriber density was associated with increase in antibiotics prescribed (17). As evident in the interviews, many patients perceive antibiotics to be a “standard-of-care”; and physicians feel that withholding it may result in patient dissatisfaction which can affect the income generating capacity of the doctor.

Apart from larger systemic issues, there are a number of local issues which can thwart the attempts to ensure an effective AMS programme in smaller hospitals. Presence of a local champion, preferably a physician who has got wide support and carries the respect of his/her colleagues, has been shown to be a very effective in the success of any initiative to rationalize antibiotic use (18). A robust microbiology laboratory is also necessary for a functional AMS programme. The communication between the microbiology lab and the treating physicians should be streamlined and there should be attempts to fast-track the delivery of results. The increase in trust between the lab and the physicians can make the AMS initiatives more effective (19). Among the seven hospitals that partnered with us in the project, only two had functional microbiology facilities. Other hospitals outsource the culture specimens to standalone labs, thereby increasing the turn-around time and costs. Traditionally, microbiology functions like blood cultures have been underutilized in LMIC contexts due to various reasons (20). The lack of facilities or inadequate number of specimens will make it extremely difficult to have a facility specific antibiogram and give recommendations about standard treatment guidelines.

The regulatory framework in many LMICs are not as effective as those in high income countries. This creates a situation where pharmaceutical companies can engage in aggressive promotional practices to ensure maximization of sales. There can be perverse incentives for prescribing/selling more antibiotics which has shown to have a definite bearing on the levels of antibiotic resistance (21). There is also a need to constantly upgrade the knowledge and skills of the prescribers regarding antimicrobial usage (22). Systemic improvements which can make the prescribers undergo regular training programmes on antimicrobial usage can possibly bridge the gap between knowledge and practice.

Antibiotic resistance warrants comprehensive action in sectors that are users of antibiotics, and healthcare facilities form one of the most important stakeholder. AMS in resource-limited setting is going to be a challenge, especially in terms of financing, access to technologies and capacity building. Political and regulatory willpower of international partnerships should be effectively harnessed for designing solutions for LMIC contexts (23). Also, models for stewardship from high income countries should undergo an adaptation process before they are introduced in low-resource settings. There should be a system to evaluate the financial and administrative feasibility of the AMS interventions, before they are rolled out in LMICs (24).

## Data Availability Statement

The datasets generated for this study are available on request to the corresponding author.

## Ethics Statement

The studies involving human participants were reviewed and approved by Institutional Review Board, Christian Medical College, Vellore, Tamil Nadu, India. The patients/participants provided their written informed consent to participate in this study.

## Author Contributions

PM, JR, and SC were involved in the conceptualization of the study. PM conducted the interviews, analyzed the data, and prepared the manuscript. JR and SC reviewed it. All authors contributed to the article and approved the submitted version.

## Conflict of Interest

The authors declare that the research was conducted in the absence of any commercial or financial relationships that could be construed as a potential conflict of interest.

## References

[1] Alsaeed A Blondeau JM. Antibiotic resistance in hospitals. Future Microbiol. (2015) 10:303–7. 10.2217/fmb.15.10 25812451

[2] Mulvey MR Simor AE. Antimicrobial resistance in hospitals: how concerned should we be? CMAJ Can Med Assoc J Assoc Medicale Can. (2009) 180:408–15. 10.1503/cmaj.080239 19221354 PMC2638041

[3] Dyar OJ Huttner B Schouten J Pulcini C ESGAP (ESCMID Study Group for Antimicrobial stewardshiP) What is antimicrobial stewardship? Clin Microbiol Infect Off Publ Eur Soc Clin Microbiol Infect Dis. (2017) 23:793–8. 10.1016/j.cmi.2017.08.026 28882725

[4] Lee BR Goldman JL Yu D Myers AL Stach LM Hedican E. Clinical impact of an antibiotic stewardship program at a children's hospital. Infect Dis Ther. (2017) 6:103–13. 10.1007/s40121-016-0139-5 27913975 PMC5336414

[5] Timbrook TT Hurst JM Bosso JA. Impact of an antimicrobial stewardship program on antimicrobial utilization, bacterial susceptibilities, and financial expenditures at an academic medical center. Hosp Pharm. (2016) 51:703–11. 10.1310/hpj5109-703 27803499 PMC5080988

[6] Shah N Joshi A Ganguly B. Impact of antibiotic stewardship program on prescribing pattern of antimicrobials in patients of medical intensive care unit. J Clin Diagn Res JCDR. (2017) 11:FC11–5. 10.7860/JCDR/2017/27171.10237 28892925 PMC5583932

[7] Lonks JR. Infection Control and Antimicrobial Stewardship. R I Med J. (2018) 101:35–7. 29857604

[8] MacDougall C Polk RE. Antimicrobial stewardship programs in health care systems. Clin Microbiol Rev. (2005) 18:638–56. 10.1128/CMR.18.4.638-656.2005 16223951 PMC1265911

[9] Stenehjem E Hyun DY Septimus E Yu KC Meyer M Raj D. Antibiotic Stewardship in Small Hospitals: Barriers and Potential Solutions. Clin Infect Dis Off Publ Infect Dis Soc Am. (2017) 65:691–6. 10.1093/cid/cix407 28472291

[10] Van Dijck C Vlieghe E Cox JA. Antibiotic stewardship interventions in hospitals in low-and middle-income countries: a systematic review. Bull World Health Organ. (2018) 96:266–80. 10.2471/BLT.17.203448 29695883 PMC5872012

[11] Cox JA Vlieghe E Mendelson M Wertheim H Ndegwa L Villegas MV. Antibiotic stewardship in low- and middle-income countries: the same but different? Clin Microbiol Infect Off Publ Eur Soc Clin Microbiol Infect Dis. (2017) 23:812–8. 10.1016/j.cmi.2017.07.010 28712667

[12] Timbrook T Maxam M Bosso J Antibiotic discontinuation rates associated with positive respiratory viral panel and low procalcitonin results in proven or suspected respiratory infections. Infect Dis Ther. (2015) 4:297–306. 10.1007/s40121-015-0087-5 26342921 PMC4575297

[13] Sexton DJ Moehring RW. Implementation of antimicrobial stewardship programs in small community hospitals: recognizing the barriers and meeting the challenge. clin Infect Dis Off Publ Infect Dis Soc Am. (2017) 65:697–8. 10.1093/cid/cix409 28472293

[14] Bishop J Kong DC Schulz TR Thursky KA Buising KL. Meeting the challenge for effective antimicrobial stewardship programs in regional, rural and remote hospitals - what can we learn from the published literature? Rural Remote Health. (2018) 18:4442. 10.22605/RRH4442 29792036

[15] Gebretekle GB Haile Mariam D Abebe W Amogne W Tenna A Fenta TG. Opportunities and barriers to implementing antibiotic stewardship in low and middle-income countries: Lessons from a mixed-methods study in a tertiary care hospital in Ethiopia. PLoS ONE. (2018) 13:e208447. 10.1371/journal.pone.0208447 30571688 PMC6301706

[16] Morgan DJ Okeke IN Laxminarayan R Perencevich EN Weisenberg S. Non-prescription antimicrobial use worldwide: a systematic review. Lancet Infect Dis. (2011) 11:692–701. 10.1016/S1473-3099(11)70054-8 21659004 PMC3543997

[17] Klein EY Makowsky M Orlando M Hatna E Braykov NP Laxminarayan R. Influence of provider and urgent care density across different socioeconomic strata on outpatient antibiotic prescribing in the USA. J Antimicrob Chemother. (2015) 70:1580–7. 10.1093/jac/dku563 25604743 PMC4398474

[18] Aagaard EM Gonzales R Camargo CA Auten R Levin SK Maselli J. Physician champions are key to improving antibiotic prescribing quality. Jt Comm J Qual Patient Saf. (2010) 36:109–16. 10.1016/S1553-7250(10)36019-3 20235412

[19] Bouza E Muñoz P Burillo A. Role of the Clinical Microbiology Laboratory in Antimicrobial Stewardship. Med Clin North Am. (2018) 102:883–98. 10.1016/j.mcna.2018.05.003 30126578

[20] Teerawattanasook N Tauran PM Teparrukkul P Wuthiekanun V Dance DAB Arif M. Capacity and utilization of blood culture in two referral hospitals in Indonesia and Thailand. Am J Trop Med Hyg. (2017) 97:1257–61. 10.4269/ajtmh.17-0193 28722626 PMC5637610

[21] Edwards SE Morel CM Busse R Harbarth S. Combatting antibiotic resistance together: how can we enlist the help of industry? Antibiotics. (2018) 7:111. 10.3390/antibiotics7040111 30567308 PMC6315850

[22] Asante KP Boamah EA Abdulai MA Buabeng KO Mahama E Dzabeng F. Knowledge of antibiotic resistance and antibiotic prescription practices among prescribers in the Brong Ahafo Region of Ghana; a cross-sectional study. BMC Health Serv Res [Internet]. (2017) 17:2. 10.1186/s12913-017-2365-2 28633631 PMC5477684

[23] Safdar N Anderson DJ Braun BI Carling P Cohen S Donskey C. The evolving landscape of healthcare-associated infections: recent advances in prevention and a road map for research. Infect Control Hosp Epidemiol. (2014) 35:480–93. 10.1086/675821 24709716 PMC4226401

[24] Bebell L Muiru A. Antibiotic use and emerging resistance-how can resource-limited countries turn the tide? Glob Heart. (2014) 9:347–58. 10.1016/j.gheart.2014.08.009 25667187 PMC4369554

