# Supplementary material for: Challenges in Implementing Antimicrobial Stewardship Programmes at Secondary Level Hospitals in India: An Exploratory Study
Source: Front Public Health. 2020 Sep 18;8:493904. doi: 10.3389/fpubh.2020.493904 (PMC7531198; doi:10.3389/fpubh.2020.493904)
Supplement: Supplementary file 1 [file Presentation_1.pdf]

An exploratory study to understand the challenges and barriers of implementing antibiotic stewardship measures: Multicenter and Multilevel hospitals.

Objective: To understand the barriers and challenges encountered in the process of implementing antibiotic stewardship measures in outpatient and inpatient settings of hospitals managed by Faith Based Organisations (FBOs) in India.

Topic Guide: In-depth interview (Physician or pharmacist leader, responsible for antibiotic stewardship)

1. Introduction

Aims: To introduce the participant to the study and create a satisfactory level of rapport with him/her.

- Introduction to researcher and research group
- Study topic and supporting agencies
- Explanation of the objectives of the study
- Explain confidentiality and anonymity
- Explain recording, length (30 mins to 45 mins) and nature of discussion, outputs/reporting and data storage issues
- Go through consent issues explaining that they may withdraw at any time from interview as whole, and do not have to answer any questions they prefer not to; position on disclosure
- Check whether they have any questions
- Check that they are happy to continue

2. Background

Aims: To get participant talking and to find out contextual information about his/her current employment

- Demographic characteristics (age, gender, education, state in India where he/she works)
- Working status (years of experience, type of hospital, place of work-city/town/interior village)
- Educational qualifications (Medical, pharmacy or public health qualifications)
- Size of the hospital (staff, outpatient load, inpatient beds, microbiology facilities, hospital information system and ICUs)

3. **Experiences with antibiotic use before stewardship measures were implemented**

Aims: To understand about their experiences with antibiotic use and Antimicrobial Resistance

- Use of antibiotics in hospital settings, in last 5 years
  - increase/ decrease/plateau
  - reason for this type of graph (commercial considerations, fear of litigation, increase in outbreaks, seasonality of diseases, government regulations, characteristics of the place of service etc)
- Usage pattern (increase or decrease) of antibiotic products In-patient and intensive care settings, in the last 5 years
  - Type of antibiotics used maximally
  - Possible reasons
    - ➢ If decrease in use reported, explore the possibilities of the centre having an antibiotic protocol, some kind of antibiotic audit, cost consideration, change in patient profile, change in doctor profile (in terms of qualification/ years of experience) etc.
    - ➢ If increase, consider possibilities like expansion of facilities, new microbiology facilities, any possibility of spurious increase, disease epidemiology of the region, commercial considerations, change in administration etc.
- Usage pattern (increase or decrease) of antibiotic products Out-patient settings, in the last 5 years
  - Type of antibiotics used maximally
  - Possible reasons
    - ➢ If decrease in use reported, explore the possibilities of the centre having an antibiotic protocol, training of doctors, feedback from pharmacy, cost consideration etc.
    - ➢ If increase, consider possibilities like fear of litigation, disease epidemiology of the region, commercial considerations, promotional benefits etc.

4. **Antibiotic Stewardship measures implemented at your hospital**

Aims: To understand about the various antibiotic stewardship measures implemented at the healthcare facility

- Types of stewardship measures planned
  - Briefly explain about the planned measures that were implemented

- Challenges faced (whether there was room for healthy discussions, whether the stakeholders were receptive, was the process democratic etc.)
    - Any alternative methods discussed during the planning stage
- Implementation process
    - Personal rating about implementation
        - areas implemented (In-patient, out-patient or ICU settings)
        - level of success
    - Challenges faced
        - Level of co-operation from pharmacists and laboratory personnel
        - Attitude of administrative personnel and hospital management
        - Inter-personal conflicts during implementation process
        - Attitude of other doctors
        - Response from patients
    - Possible reasoning for the challenges faced (pressure from the pharma companies, hurting the doctor's ego, fear of losing patients' confidence, doctors do not like to be dictated etc.)
- Impact of stewardship measures on antibiotic prescriptions
    - Increase/Decrease/Plateau of overall quantity of antibiotics prescribed
        - Change in quantity noticed
        - Place of change (In-patient, Out-patient or ICU settings)
        - Quantum of change proportional to the efforts invested in stewardship measures
    - Change in the type of antibiotic being prescribed
        - Older or newer generation antibiotics
        - Place of change (In-patient, Out-patient or ICU settings)
    - Change in attitude of personnel
        - Positive or Negative attitude of physicians and other personnel
        - Support for other planned stewardship measures.
    - Any untoward incident, attributed to antibiotic stewardship measures
        - Type of incident or incidents (Complication, morbidity, mortality)
        - Possible reasons for attributing to stewardship measures
- Other challenges in the implementation process
    - Lack of infrastructural facilities (like microbiology lab for culture & sensitivity, hospital information system, computerized pharmacy etc)
    - Lack of support from patients (demand for antibiotics from patients, over-the-counter antibiotic abuse etc)

5. **Future**

Aim: to find out about their reflections on the future of antibiotic stewardship in their respective hospitals

- Personal conviction with respect to implementing this program (motivated or not, conducting it for the sake of doing it, possible reasons etc)
- Do they expect their hospital to take forward the stewardship program
    - the possible reasons for this decision
- Any modifications they would like to make to the program they have currently implemented
- Any other points they would like to add

6. **In conclusion**

Aim: to reiterate confidentiality and to ask permission to archive participant's transcript for research purposes

- Thank the participants for their time. Reiterate that the discussion findings will remain confidential. Tell them that they are welcome to contact members of the study team to ask questions at a later date if they wish
- Ask participants for permission to archive the transcript of the interview. Explain that it will be fully anonymised and will allow other researchers to use the information they have given for research purposes.
